# Public perceptions and knowledge of cholesterol management in a multi-ethnic Asian population: A population-based survey

**DOI:** 10.1371/journal.pone.0256218

**Published:** 2021-08-13

**Authors:** Chiw Yeh Lim, Jien Sze Ho, Zijuan Huang, Fei Gao, Swee Yaw Tan, Woon Puay Koh, Terrance Chua, Lip Ping Low, Huay Cheem Tan, Sungwon Yoon

**Affiliations:** 1 Department of Cardiology, National Heart Centre Singapore, Singapore, Singapore; 2 National Heart Research Institute Singapore, National Heart Centre Singapore, Singapore, Singapore; 3 Health Services and Systems Research, Duke-NUS Medical School, Singapore, Singapore; 4 Healthy Longevity Translational Research Programme, Yong Loo Lin School of Medicine, National University of Singapore, Singapore, Singapore; 5 Low Cardiology Clinic, Mount Elizabeth Medical Centre, Singapore, Singapore; 6 Department of Cardiology, National University Heart Centre, Singapore, Singapore; 7 Regional Health System, Singapore Health Services, Singapore, Singapore; Medizinische Universitat Graz, AUSTRIA

## Abstract

**Introduction:**

Cardiovascular diseases (CVDs) are the leading cause of mortality worldwide. Hyperlipidemia is one of the important modifiable risk factors for CVDs. Raising public awareness of CVD risks is an important step in reducing CVD burdens. In this study, we aimed to assess public awareness and knowledge of cholesterol and its management in a multiethnic Asian population.

**Methods:**

We recruited 1000 participants from three major ethnic groups for this nationwide population-based survey. A structured questionnaire was used to collect socio-demographics, knowledge of cholesterol and cholesterol-lowering medications. Univariate and multivariate analyses were conducted to identify factors associated with good knowledge on cholesterol and its management.

**Results:**

Of the participants, 65% thought that high cholesterol produces symptoms and that lifestyle modification would be as effective as medication at lowering cholesterol. Nearly 70% believed that long term statin could lead to kidney or liver damage, and 56% thought that statin was associated with higher risk of cancer. A third saw herbal medicine or supplements as healthier and safer. About 45% believed that statin therapy should not be taken long term and that one could stop taking cholesterol medication when cholesterol is under control. Malays were more likely to have poor knowledge (adjusted OR 0.68; 95% CI 0.47–0.98; P = 0.039) compared to Chinese. Participants with intermediate education were more likely to have good knowledge of cholesterol and its management (adjusted OR 1.67; 95% CI 1.11–2.51; P = 0.013) compared to those with primary education.

**Conclusion:**

Public knowledge and awareness of high cholesterol and its management remains poor in Asian multi-ethnic population. Understanding gaps in public knowledge can inform the implementation of health promotion programs to effectively raise awareness of cholesterol and its management.

## Introduction

Cardiovascular diseases (CVDs) which include coronary heart disease and cerebrovascular disease are the leading cause of mortality worldwide [1, 2]. CVDs are responsible for an estimated 17.9 million deaths each year, an estimated 31% of all deaths worldwide [3]. Four out of five CVD deaths are attributed to heart attacks and strokes [3]. Premature death in people under 70 years of age also makes up one third of these deaths [3]. In addition to being the leading cause of death, ischemic heart disease and stroke are the top three global causes of disability-adjusted life years in 2019 [1]. In Singapore, CVDs (both ischemic heart diseases and cerebrovascular diseases) are the second highest cause of mortality, accounting for 24.6% of deaths in 2019 [4].

Hyperlipidemia is one of the important modifiable risk factors for CVDs. Lowering lipid levels with HMG-CoA reductase (3-hydroxy-3-methyl-glutaryl-coenzyme A reductase) inhibitors, or statins, is a proven effective therapy to reduce mortality. Multiple randomized controlled trials have unequivocally proven that statin therapy reduces the risk of major vascular events by about one-quarter (25%) for 1 mmol/L reduction in LDL cholesterol each year after the first year [5]. Effective statin dose can reduce LDL by 2 mmol/L and could reduce close to half the patient’s risk of heart attacks and strokes [5]. Despite the many benefits, adherence to statin therapy for primary and secondary prevention is low [6, 7]. Furthermore, it is of concern that exaggerated claims about side-effects of statin may be responsible for its low adherence among individuals at increased risk of cardiovascular events.

Educating the general public with adequate knowledge and awareness of CVD risk factors is an important step in reducing CVD burdens. Although knowledge does not always result in the adoption of preventive behaviour [8–10], knowledge is crucial to building necessary skills to apply what is learned and making an informed decision. Studies have shown that there are gaps in the level of understanding of cholesterol and its treatment [11, 12]. However, much of the existing literature on cholesterol knowledge has focused on patients with hyperlipidemia [13–15]. Population-based research on the general public’s knowledge and misconception of cholesterol is scarce, particularly in Asian population [11, 12]. In this nationwide multi-ethnic population survey, we aimed to assess public awareness and knowledge of cholesterol and its management, and identify factors associated with the level of knowledge.

## Methods

### Sample and setting

We engaged a professional market survey company to conduct a nationwide population-based survey. The residential areas in Singapore were divided into 26 subzones. The participants sample selection was stratified first by subzone then by housing type (public housing, landed property, condominium and shop house). Random sampling of households to approach was carried out. Our study included Singapore citizens and permanent residents between the ages of 21 and75. We sampled an equal number of individuals from the three major racial groups in Singapore (i.e. Chinese, Malays and Indians). A total of 1000 members were recruited for the survey. Door to door survey was conducted from April 2018 to May 2018. The survey was administered in one of the four national languages: English, Mandarin, Malay or Tamil. Only one respondent was interviewed per household. This study was approved by National University of Singapore’s Institutional Review Board (S-17-256E). Verbal consent was obtained before proceeding with the survey.

### Instrument

A structured questionnaire, pilot-tested with ten respondents, was used to collect demographic data including age, gender, race, cardiovascular risk factor, education, employment status. Pilot participants were selected based on age, gender and language used for daily communication. Face-to-face cognitive interviews were carried out in the patient waiting areas of the public hospitals. Feedback was collected to assess content validity of the questionnaire items. Modifications were subsequently made. Cardiovascular risk factors were categorized into ’yes’ if any of the following was present (heart disease, stroke, hypertension, diabetes, hyperlipidemia) and ’no’ if none was present. Education was classified into ‘low’ (primary school or below), ‘intermediate’, or ‘high’ (university education and above). Employment status was classified into ‘working’ (full time employed, part time employed, self-employed, employers), ‘retired/homemakers’ (retirees and homemakers), ‘others’ (National servicemen, students, unemployed).

Participants were asked questions on possible symptoms of high cholesterol, safety of cholesterol-lowering herbal remedies versus western medications, effectiveness of diet and lifestyle in cholesterol-lowering versus medication, and their knowledge of cholesterol-lowering medications. Responses were recorded as true, false or don’t know. A score was generated for the knowledge of cholesterol and its management with one point given for each correct answer. Subjects were classified into a good knowledge group (3–8 correct answers) and a poor knowledge group (0–2 correct answers).

### Statistical analysis

Data was summarized and presented using descriptive statistics: counts and percentages for categorical variables, means and standard deviations for continuous variables. Results weighted by race on knowledge scores of cholesterol and their associations with socio-demographic variables were reported. Logistic regressions were performed based on weighted results on race. The multivariate analysis included variables that were significant in the univariate analysis. Statistical analysis was performed using STATA version 15 with statistical significance set at p < 0.05.

## Results

### Socio-demographic characteristics

Of the 1,000 participants who were interviewed, 479 (47.9%) were male and 521 (52.1%) were female. Three hundred and fifty-seven (35.7%), 321 (32.1%) and 322 (32.2%) were Chinese, Malay and Indian, respectively. Mean age was 45 years. More than two thirds of participants had an intermediate level of educational attainment. Two thirds of respondents were in the workforce. There was no missing data. The majority of participants (86.2%) did not have a cardiovascular risk factor, with 9.6% having one cardiovascular risk factor and 4.2% having two or more risk factors. Seventy (7%) had hyperlipidemia. Socio-demographic characteristics of the sample are summarized in Table 1.

**Table 1:** Demographics of survey respondents (n = 1,000).

Variable	Category	Whole Cohort	Chinese (n = 357)	Malay (n = 321)	Indian (n = 322)
Age, years		45 ± 14.5*	47 ± 14.6	44 ± 14.6	45 ± 14.1
Age (years)	20–39	385	123 (34.5)**	138 (43.0)	124 (38.5)
	40–59	413	142 (39.8)	130 (40.5)	141 (43.8)
	60–75	202	92 (25.8)	53 (16.5)	57 (17.7)
Gender	Female	521	186 (52.1)	168 (52.3)	167 (51.9)
	Male	479	171 (47.9)	153 (47.7)	155 (48.1)
Educational level	Low education (primary school or less)	194	65 (18.2)	70 (21.8)	59 (18.3)
	Intermediate education	679	216 (60.5)	234 (72.9)	229 (71.1)
	High education (university or above)	127	76 (21.3)	17 (5.3)	34 (10.6)
Employment	Employed full time	551	197 (55.2)	177 (55.1)	177 (55.0)
	Employed part time	78	22 (6.2)	28 (8.7)	28 (8.7)
	Employer/self employed	36	19(5.3)	8 (2.5)	9 (2.8)
	Unemployed looking for work	13	1 (0.3)	7 (2.2)	5 (1.6)
	Not working not looking for work	39	11 (3.1)	10 (3.1)	18 (5.6)
	Retired	94	42(11.8)	27 (8.4)	25 (7.8)
	Homemaker	141	49 (13.7)	45(14.0)	47 (14.6)
	Full time National Service	10	4(1.1)	6 (1.9)	0 (0)
	Full time student	38	12 (3.4)	13 (4.1)	13 (4.0)
Cardio-vascular risk factor	Heart disease	22	10 (45.45)	5 (22.72)	7 (31.81)
	Stroke	9	2 (22.22)	3 (33.33)	4 (44.44)
	Hypertension	50	26 (52.0)	11 (22.0)	13 (26.0)
	Diabetes	51	15 (29.41)	18 (35.29)	18 (35.29)
	Hyperlipidaemia	70	41 (58.57)	14 (20.0)	15 (21.42)

*mean ± standard deviation.

**frequency (percent).

### Public perception and knowledge of cholesterol and its management

Table 2 shows the statements from the questionnaire and participants’ responses. All the statements were false statements. Nearly two thirds (65%) of respondents thought that people with high cholesterol usually have symptoms such as breathlessness or chest pain. Sixty eight percent of respondents believed that long term statin therapy could lead to kidney or liver damage, and more than half of the respondents (56%) had the misconception that statin was associated with higher risk of cancer. The majority of respondents (65%) thought that lifestyle modification such as diet and exercise would be as effective as medication at lowering cholesterol. In terms of the safety of alternative medicine, one third of respondents thought that herbal medicine or supplements are healthier and safer as compared to Western prescribed medication. A substantial proportion of respondents (45%) believed that statin therapy should not be taken long term. A considerable percentage of respondents (45%) felt that one could stop taking cholesterol medication when cholesterol is under control.

**Table 2:** Level of knowledge on cholesterol and its management (n = 1,000).

Statement	Participant Response n (%)		
(All statements are false statements)	True	False	Don’t know/unsure
People with high cholesterol usually have symptoms such as breathlessness or chest pain.	646 (65%)	192 (19%)	162 (16%)
To control cholesterol, it is healthier and safer to take herbal medicine or supplement than taking prescribed Western medication.	323 (32%)	343 (34%)	334 (34%)
Diet and exercise are equally effective at lowering cholesterol as medication.	647 (65%)	176 (18%)	177 (18%)
Long-term use of medication for high cholesterol such as statins can damage the kidney and liver.	680 (68%)	100 (10%)	220 (22%)
Taking long-term cholesterol medication such as statins is associated with a higher risk of cancer.	557 (56%)	180 (18%)	263 (26%)
It is not safe to stop long-term cholesterol medication such as statins once started on the medication.	557 (56%)	139 (14%)	304 (30%)
Long-term cholesterol medication such as statins can be stopped once cholesterol is under control.	451 (45%)	254 (25%)	295 (30%)
Statin should not be taken long term.	447 (45%)	149 (15%)	404 (40%)

### Factors associated with public knowledge of cholesterol and its management

Twenty-six participants who answered ‘don’t know/unsure’ to all 8 statements were excluded from logistic regression analysis. In the multivariable logistic regression model, Malays were more likely to have poor knowledge (adjusted OR 0.68; 95% CI 0.47–0.98; P = 0.039) as compared to Chinese. Participants with intermediate education were more likely to have good knowledge (adjusted OR 1.67; 95% CI 1.11–2.51; P = 0.013) as compared to those with primary education. The logistic regression model is shown in Table 3.

**Table 3:** Factor associated with good knowledge.

Factors	Good knowledge (n = 729)	Poor knowledge (n = 245)	Unadjusted		Adjusted	
			OR (95% CI)	P-value	OR (95% CI)	P-value
**Age group**						
20–39	289 (39.6%)	84 (34.3%)	1.0		1.0	
40–59	307 (42.1%)	97 (39.6%)	1.06 (0.75–1.50)	0.74	1.20 (0.83–1.71)	0.33
60–75	133 (18.2%)	64 (26.1%)	0.66 (0.45–0.96)	0.030	0.87 (0.54–1.42)	0.58
**Gender**						
Male	340 (46.6%)	125 (51.0%)	1.0			
Female	389 (53.4%)	120 (49.0%)	1.17 (0.87–1.57)	0.29		
**Race**						
Chinese	273 (37.5%)	79 (32.2%)	1.0		1.0	
Malay	222 (30.5%)	93 (38.0%)	0.69 (0.48–0.99)	0.041	0.68 (0.47–0.98)	0.039
Indian	234 (32.1%)	73 (29.8%)	0.93 (0.56–1.54)	0.77	0.90 (0.54–1.50)	0.68
**Employment**						
Working	498 (68.3%)	148 (60.4%)	1.0		1.0	
Retired/homemakers	157 (21.5%)	74 (30.2%)	0.68 (0.49–0.94)	0.020	0.90 (0.59–1.37)	0.62
Others	74 (10.2%)	23 (9.4%)	1.24 (0.70–2.20)	0.46	1.42 (0.79–2.54)	0.24
**Education**						
Primary or less	123 (16.9%)	67 (27.4%)	1.0		1.0	
Intermediate	507 (69.6%)	151 (61.6%)	1.85 (1.30–2.65)	0.001	1.67 (1.11–2.51)	0.013
University or above	99 (13.6%)	27 (11.0%)	2.03 (1.25–3.28)	0.004	1.63 (0.94–2.83)	0.085
**Have CVD factor**						
No	626 (85.9%)	212 (86.5%)	1.0			
Yes	103 (14.1%)	33 (13.5%)	1.18 (0.78–1.76)	0.43		

OR = 1.0 is the reference category.

Three factors were statistically significant in univariate analysis, but were not statistically significant after multivariate analysis. These were age group between 60–75 (unadjusted OR 0.66; 95% CI 0.45–0.96; P = 0.03; adjusted OR 0.87; 95% CI 0.54–1.42; P = 0.58), retired/homemakers (unadjusted OR 0.68; 95% CI 0.49–0.94; P = 0.02; adjusted OR 0.90; 95% CI 0.59–1.37; P = 0.62) and university or above education (unadjusted OR 2.03; 95% CI 1.25–3.28); P = 0.004; adjusted OR 1.63; 95% CI 0.94–2.83; P = 0.085).

## Discussion

This study examined the knowledge of the general public on cholesterol and its management. Our findings suggested that overall public knowledge on cholesterol remains very low. In our study, 85% did not know that cholesterol medication should be taken long term and 81% did not know that high cholesterol per se does not produce any symptom. This finding echoes prior literature. For example, a study by Nash et al. [11] in United States reported that 51% of healthy adults aged 40 years and above did not know their own cholesterol level while 53.1% overestimated the cholesterol goal for a healthy adult. Similar findings were observed in patients with high cholesterol where better knowledge could have been anticipated. Consoli et al. [13] found that one in four hypercholesterolemic patients in France thought that they could sense an increase in their blood cholesterol level without needing to see the blood test results and only half of patients knew the acceptable upper limit for blood cholesterol. In a global survey of 1,547 patients with high cholesterol in ten countries (Belgium, Brazil, Denmark, Finland, France, Mexico, Portugal, Singapore, South Korea and the United Kingdom) [14], around 60% heard of ‘bad’ (low-density lipoprotein) or ‘good’ (high-density lipoprotein) cholesterol, but 19% of patients were unable to specify any consequences of high cholesterol. A similar lack of understanding of cholesterol was also observed in Asian patients. A survey of 853 patients with high cholesterol in India found that 57% did not know that they had high cholesterol while 22.4% did not understand the reason for taking cholesterol lowering medication [15]. Lack of knowledge of one’s cholesterol level can cause undiagnosed hypercholesterolemia. In our study, the self-reported rate of hyperlipidemia is 7%. Data derived from a national sample of 19,489 adults in Korea found that in younger adults (<50 years old) who had high cholesterol, only 8% were aware of the condition [16].

In our study, a large proportion of respondents (65%) thought that diet and exercise alone were as effective as medication at lowering cholesterol. In addition, the vast majority (68%) had misconceptions about the association of statin therapy with liver and kidney failure and higher risk of cancer. This finding is consistent with previous literature [13] that lipid lowering drugs were commonly believed to have drawbacks and that only 45% of hypercholesterolemic patients believed that drug therapy was the most effective way of lowering blood cholesterol. A qualitative analysis [17] of social media posts about statin identified that beneficial effects of statins accounted for 4.6% of the total posts while beliefs about harms or medical mistrust accounted for 20% of the posts. Posts on harm were either general reference to statin as “dangerous” or “poisonous”, or adverse events such as dementia, liver failure and mortality. Our finding, together with previous literature, suggests that there is a need to improve the knowledge of cholesterol in the general public and patients with high cholesterol.

Our study identified race as a factor that influences knowledge on cholesterol and belief on statin. Malays were more likely to have less knowledge of cholesterol and more misconceptions about cholesterol management compared to Chinese. The difference in patient perceptions of statin efficacy and safety by race was demonstrated in a previous study [18]. Nanna et al. [18] found that African Americans were less likely than whites to believe that statins are safe (36% vs. 57%) and effective (70% vs. 74%). African Americans also had lower knowledge of personal and target cholesterol compared to whites [19]. Our findings also suggested that education level was associated with knowledge on cholesterol. People of primary education level were more likely to have poor knowledge compared with those of intermediate education level. This association was not evident in the group with high education level, which could be due to much smaller numbers in the high education group leading to the result being insignificant. Our finding is in line with a previous study in France [13] that knowledge of cholesterol differed markedly according to the patient’s educational level with hypercholesterolemic patients with lower education having poorer knowledge. Our findings demonstrate that health promotion efforts to increase personal awareness of cholesterol and its management should target subgroups of population such as the Malay and people of lower education.

In addition to guidelines for clinical management of hyperlipidemia [20–23], more efforts should equally focus towards prevention. The World Health Organization (WHO) non-communicable disease Global Action Plan 2013–2020 emphasizes the role of cholesterol management to prevent CVD event [24]. The global action plan aims to lower the mortality of four major diseases (including CVD) by 25% and provide appropriate medications in ≥50% of patients to prevent heart attack and stroke [24]. The WHO recommends prescription of statins along with antiplatelet, antihypertensive medications, highlighting the importance of lipid management in the prevention of first heart attack or stroke in adults aged 40–79 years [24]. In US, *Healthy People* initiated by the U.S. Department of Health and Human Services since 1980 sets measureable objectives for improving health and well-being nationwide. Objectives of the *Healthy People* include increase in the proportion of patients going for blood cholesterol evaluation, reduction in the proportion of patients with high cholesterol and reduction in mean total cholesterol levels by 2020 [25]. In 2017, 88% of adult population had their blood cholesterol screening compared to 74.6% in 2008 [25]. Million Hearts® initiative which began in 2012 aims to prevent 1 million heart attacks and strokes within 5 years. It focuses on implementing evidence-based priorities and targets to keep people healthy. To optimize cardiovascular care, the program aims to increase aspirin use, blood pressure and cholesterol control and smoking cessation to 80%. In its first 5-year cycle (2012–2016), Million Hearts® had prevented an estimated 135,000 heart attacks, strokes and related acute cardiovascular events [26]. Similarly, in the UK, the English National Health Service Health Checks program started in 2009, aiming to reduce CVD mortality. The program aims to provide a routine structured clinical assessment and management for adults aged 40–74 years without pre-existing diabetes or CVD. Four years after its implementation, an analysis showed a high rate of newly identified comorbidities [27]. Of the high CVD risk attendees, 19.3% were newly prescribed statins therapy [27]. A similar approach could be considered to improve public knowledge of cholesterol and reduce the risk of developing CVD and its complications in Asian populations including Singapore.

This study has several limitations. This is a public survey and only a small proportion of participants had CVD risk factors. Therefore, we were unable to determine whether having CVD was associated with the level of cholesterol knowledge. Future research can aim at investigating the knowledge of cholesterol and management in Asian patients with hyperlipidemia. Those who participated in this survey may have been more health conscious or aware of hyperlipidemia than the general population. In addition, compared to the general Singapore population, our study population had a greater proportion of participants with intermediate educational attainment (42.1% vs 67.9%) and a lower proportion of participants with university and above educational attainment (32.4% vs 12.7%) [28], which might have affected our findings. The self-report nature of the risk factors such as hyperlipidemia and hypertension might have introduced a social desirability bias. A notable limitation to the study was that all eight statements assessing the cholesterol knowledge were false. Participants who were unsure of the answer might have chosen some statements to be true to give a ‘balanced’ answer. This may have influenced the results of the study [29, 30].

## Conclusion

Public knowledge and awareness of high cholesterol and its management remains poor in Asian multiethnic populations. Understanding gaps in public knowledge can inform the implementation of health promotion programs to effectively raise awareness of cholesterol and its management. Future interventions should focus on developing culturally tailored public health messages to close some of the racial disparity in cholesterol knowledge. Greater emphasis could also be placed on targeted educational efforts among lower education groups.

## Supporting information

S1 Dataset (XLSX)

S1 Questionnaire (DOCX)
