# Supplementary material for: Public perceptions and knowledge of cholesterol management in a multi-ethnic Asian population: A population-based survey
Source: PLoS One. 2021 Aug 13;16(8):e0256218. doi: 10.1371/journal.pone.0256218 (PMC8362937; doi:10.1371/journal.pone.0256218)
Supplement: S1 Questionnaire — (DOCX) [file pone.0256218.s002.docx]

**POPULATION-BASED HEART HEALTH SURVEY IN SINGAPORE**

**QUESTIONNAIRE**

**Medical history**

1. Have you even been told by a doctor nurse or other health professionals that you have the following conditions?

| Disease | Yes or No | | On Medication to treat.. | | Use of herbal or traditional treatment to treat | |
| --- | --- | --- | --- | --- | --- | --- |
| 22-a.  Heart disease (heart attack, heart artery blockage) | 1) Yes | 2) No | 1) Yes | 2) No | 1) Yes | 2) No |
| 22-b. Stroke | 1) Yes | 2) No | 1) Yes | 2) No | 1) Yes | 2) No |
| 22-c. Hypertension | 1) Yes | 2) No | 1) Yes | 2) No | 1) Yes | 2) No |
| 22-d. Diabetes Mellitus | 1) Yes | 2) No | 1) Yes | 2) No | 1) Yes | 2) No |
| 22-e. Hyperlipidemia  (e.g., high level of lipids such as fats, cholesterol and triglycerides in your blood) | 1) Yes | 2) No | 1) Yes | 2) No | 1) Yes | 2) No |

**Knowledge of hypercholesterolemia**

2. Please read the statement(s) below and decide each is true or false.

|  |  | 1) True | 2) False | 3) Don’t know/unsure |
| --- | --- | --- | --- | --- |
| **People with high cholesterol** | Usually have symptoms such as breathlessness or chest pain |  |  |  |
| **In controlling the cholesterol** | Taking herbal medicine/supplements is safer than taking Western medication. |  |  |  |
|  | Diet and exercise is as effective as medication in controlling cholestrol. |  |  |  |
| **Long-term cholesterol medication such as statins** | Can damage the kidney or liver. |  |  |  |
|  | Is associated with a higher risk of cancer. |  |  |  |
|  | Is not safe to stop the medicine once started. |  |  |  |
|  | Can be stopped once cholesterol is under control. |  |  |  |
|  | Statin should not be taken long term. |  |  |  |

###### DEMOGRAPHIC & SOCIOECONOMIC INFORMATION

3. Gender

1. Male
2. Female

4. Weight (kg) ___________ Height (cm) _______________

5. What is your race?

1. Chinese
2. Malay
3. Indian
4. Others

6. Year of birth ____________________

7. Employment status

1. Employed (full-time)
2. Employed (part-time)
3. Unemployed
4. Homemaker/housewife
5. Student
6. Retired

8. Current marital status

1. Currently Married
2. Divorced
3. Separated
4. Widowed
5. Never married

9. Highest education level

1. No formal education
2. Primary school
3. Secondary school
4. Junior College/polytechnic
5. University/degree/higher levels

10. Over the past 12 months, the average earnings of the household earnings **per month**

1. Below 5,000 per month
2. 5,000-10,000 per month
3. 10,000 and above per month

11. Housing Type *(No need to ask, the survey personnel will record this)*

1. HDB 1 room
2. HDB 2 or 3 room
3. HDB 4 or 5 room
4. Private condominium
5. Landed property
6. Others (specify: ______________)
